# Relación de la calidad de sueño, adherencia al tratamiento, hemoglobina glucosilada en mujeres con diabetes**

**DOI:** 10.15649/cuidarte.1996

**Published:** 2022-08-08

**Authors:** Ingrid Carolina Simental Oliva, Francisco Javier Baez Hernández, Vianet Nava Navarro, Marcela Flores Merlo, Arelia Morales Nieto, Miguel Ángel Zenteno López

**Affiliations:** 1 Instituto Mexicano del Seguro Social, Puebla, México. E-mail: simentalcaro2584@gmail.com Instituto Mexicano del Seguro Social Instituto Mexicano del Seguro Social Puebla Mexico simentalcaro2584@gmail.com; 2 Facultad de Enfermería, Benemérita Universidad Autónoma de Puebla, Puebla, México. E-mail: javier.baez@correo.buap.mx Benemérita Universidad Autónoma de Puebla Facultad de Enfermería Benemérita Universidad Autónoma de Puebla Puebla Mexico javier.baez@correo.buap.mx; 3 Facultad de Enfermería, Benemérita Universidad Autónoma de Puebla, Puebla, México. E-mail: vianet.nava@correo.buap.mx Benemérita Universidad Autónoma de Puebla Facultad de Enfermería Benemérita Universidad Autónoma de Puebla Puebla Mexico vianet.nava@correo.buap.mx; 4 Facultad de Enfermería, Benemérita Universidad Autónoma de Puebla, Puebla, México. E-mail: marcela.floresm@correo.buap.mx Benemérita Universidad Autónoma de Puebla Facultad de Enfermería Benemérita Universidad Autónoma de Puebla Puebla Mexico marcela.floresm@correo.buap.mx; 5 Facultad de Enfermería, Benemérita Universidad Autónoma de Puebla, Puebla, México. E-mail: arelia.morales@correo.buap.mx Benemérita Universidad Autónoma de Puebla Facultad de Enfermería Benemérita Universidad Autónoma de Puebla Puebla Mexico arelia.morales@correo.buap.mx; 6 Facultad de Enfermería, Benemérita Universidad Autónoma de Puebla, Puebla, México. E-mail: miguel.zenteno@correo.buap.mx Benemérita Universidad Autónoma de Puebla Facultad de Enfermería Benemérita Universidad Autónoma de Puebla Puebla Mexico miguel.zenteno@correo.buap.mx

**Keywords:** Sueño, Cumplimiento y Adherencia al tratamiento, Diabetes Mellitus Tipo 2, Mujeres, Hemoglobina A Glucada, Sleep, Treatment Adherence and Compliance, Diabetes Mellitus, Type 2, Women, Glycated hemoglobin A, Sono, Cooperação e Adesão ao Tratamento, Diabetes Mellitus Tipo 2, Mulheres, hemoglobina A glicada

## Abstract

**Introducción:**

La Diabetes Tipo 2 es considerado un problema de salud pública que afecta principalmente a las mujeres, que sumado a una mala adherencia al tratamiento terapéutico y, a una falta de calidad de sueño, aumentan la problemática de salud.

**Objetivo:**

Determinar las interrelaciones existentes entre la Calidad de Sueño, la Adherencia al Tratamiento Terapéutico y los valores de HbA1c en Mujeres con DT2, perteneciente a una comunidad de la ciudad de Puebla.

**Materiales y Métodos:**

El diseño del estudio fue de tipo descriptivo, correlacional y de corte transversal. La muestra se calculó con un nivel de significancia de .05, un coeficiente de correlación .30 y un poder estadístico del 90%, obteniendo una n=110. Los instrumentos utilizados fueron: una cédula de datos personales, el Índice de calidad de sueño de Pittsburgh (PSQI), el Cuestionario de Adherencia Terapéutica MBG (Martín-Bayarre-Grau) y el dispositivo Eclipse A1c.

**Resultados:**

Se encontró una relación negativa y significativa para la calidad de sueño con los niveles de HbA1c (rs=-.355; p=.001); no así para con la variable de adherencia al tratamiento terapéutico.

**Discusión:**

La información obtenida concuerda con otros estudios, al reafirmar de manera indirecta, las reacciones bioquimicas que ocurren durante la privación del sueño.

**Conclusiones:**

Los resultados descubiertos contribuyen al fortalecimiento científico de enfermería, orientando en la mejora de cuidados, que servirá para el diseño de intervenciones que favorezcan a la salud de las mujeres con Diabetes Tipo 2.

## Abstract (en)

**Introduction:**

Type 2 diabetes is considered a public health problem mainly affecting women, that combined with poor adherence to therapeutic treatment and poor sleep quality increase this health problem.

**Objective:**

To determine existing associations between sleep quality, adherence to therapeutic treatment and HbA1c levels in women with T2D from a community in Puebla.

**Materials and Methods:**

A cross-sectional descriptive correlational study was conducted using a sample calculated at 0.05 level of significance, 0.30 correlation coefficient and 90% statistical power to obtain n=110. Instruments used were personal data forms, Pittsburgh Sleep Quality Index (PSQI), Martín-Bayarre-Grau (MBG) Questionnaire to assess therapeutic adherence and Eclipse A1c analyzer.

**Results:**

A negative significant relationship was found for sleep quality with HbA1c levels (rs=-.355; p=.001), but not for the treatment adherence treatment variable.

**Discussion:**

Retrieved data is in accordance with other studies reaffirming indirectly all biochemical reactions occurring during sleep deprivation.

**Conclusions:**

Findings herein contribute to the strengthening of the scientific side of nursing oriented to care improvement, which will serve for the design of interventions promoting healthcare in women with type 2 diabetes.

## Resumo

**Introdução:**

A diabetes tipo 2 é considerada um problema de saúde pública que afeta principalmente as mulheres, o que somado a uma má aderência ao tratamento terapêutico e, a falta de qualidade do sono, aumenta o problema de saúde.

**Objetivo:**

Determinar as inter-relações entre a qualidade do sono, a adesão ao tratamento terapêutico e os valores de HbA1c nas mulheres com DT2, pertencentes a uma comunidade da cidade de Puebla.

**Materiais e Métodos:**

O desenho do estudo foi descritivo, correlacional e transversal. A amostra foi calculada com um nível de significância de 0,05, um coeficiente de correlação de 0,30 e um poder estatístico de 90%, obtendo-se uma n=110. Os instrumentos utilizados foram: um formulário de dados pessoais, o Índice de Qualidade de Sono de Pittsburgh (PSQI), o Questionário MBG para Avaliar Adesão Terapêutica (Martín-Bayarre-Grau) e o dispositivo Eclipse A1c.

**Resultados:**

Foi encontrada uma relação negativa e significativa da qualidade do sono com os níveis de HbA1c (rs=-.355; p=.001); este não foi o caso para a variável de aderência ao tratamento terapêutico.

**Discussão:**

As informações obtidas concordam com outros estudos, reafirmando indiretamente as reações bioquímicas que ocorrem durante a privação do sono.

**Conclusões:**

Os resultados descobertos contribuem para o fortalecimento científico da enfermagem, orientando a melhoria dos cuidados, que servirão para o desenho de intervenções que favoreçam a saúde das mulheres com diabetes tipo 2.

## Introducción

Se calcula a nivel mundial que cada seis segundos una persona muere a causa de la diabetes tipo 2 (DT2); en el año 2014 se estimó que alrededor de 422 millones de adultos en todo el mundo la padecían, lo que corresponde 1 de cada 11 personas^1^.

De acuerdo con los datos epidemiológicos de la Federación Internacional de Diabetes^2^, en América del Norte y el Caribe existen aproximadamente 48 millones de personas con DT2; la situación en México no es diferente, con base en la Encuesta Nacional de Salud y Nutrición^3^, se encontró que la prevalencia de DT2 en el país pasó del 9.7% en el año 2012 a 11.4% en el año 2018, con mayor prevalencia en mujeres de 40 a 79 años edad con un 87.9%. Asimismo, se reportó el número de casos por entidad federativa en el que se estima que 9% del Estado de Puebla padece esta enfermedad; de acuerdo al boletín epidemiológico de la Secretaria de la Salud^4^, en la semana número 44 del año 2020, se reportaron 551 casos de personas con DT2, de los cuales 56.84% fueron mujeres.

Con base al planteamiento epidemiológico descrito, la DT2 es considerada un problema de salud pública, debido a su alto índice de mortalidad y morbilidad; mismas que principalmente son causadas a consecuencias de un mal control glucémico debido entre otras cosas, a una mala adherencia al tratamiento terapéutico, que van desde el punto de vista fisiológico a complicaciones microvasculares y macrovasculares^1^; sin embargo, se dejan de lado algunas otras alteraciones como las cognitivas, que conllevan a la falta de concentración, cambios de humor, habilidad para realizar algunas actividades y además recientemente se ha encontrado que la calidad de sueño se relaciona sobre los procesos metabólicos, lo que aumenta la problemática de salud^5^.

La calidad de sueño implica aspectos de eficacia subjetiva, latencia, duración y vigor habitual, así como disminuciones o nulas perturbaciones del sueño, uso de medicamentos hipnóticos y disfunción diurna^6^^,^^7^. En este sentido, se han encontrado hallazgos contradictorios en países bajos, Tailandia, India, Japón e Irán, acerca de la relación significativa entre la calidad de sueño y los niveles HbA1c, en personas con DT2^8^^-^^12^, como reflejo de la adherencia al tratamiento terapéutico que tiene el individuo, situación que pone en evidencia un vacío de conocimiento sobre esta problemática de salud.

Lo anterior pudiera deberse a los diferentes contextos geográficos y sociodemográficos en los que se han realizado dichas investigaciones, así como al no considerar variables como la adherencia al tratamiento terapéutico, entendida como la implicación activa y voluntaria que tiene la persona, para el cumplimiento de las prescripciones médicas, que conllevan al control glucémico de la DT2^13^^,^^14^, hace suponer la importancia que tiene dicha variable, para ser un determinante junto con la calidad de sueño, sobre los valores de HbA1c; por lo tanto, es necesario abordar estas variables en su conjunto, para poder contribuir al cuerpo de conocimiento, desde la perspectiva de enfermería, dado que es una disciplina que está en una posición ideal para favorecer con la descripción de esta situación de salud y, de manera particular en el género el femenino, que es el que más prevalece la DT2.

Dado que la calidad del sueño, la adherencia al tratamiento terapéutico y la alteración de los valores de HbA1c no son variables que afectan solamente los componentes fisiológicos de la persona, sino también los aspectos psíquicos, emocionales y sociales^15^. Hace pertinente que los profesionales de la salud aborden este tipo de estudio, ya que permitirá conocer más sobre este fenómeno de interés, que en el caso específico de enfermería podría contribuir a nuevas estrategias de cuidados en la práctica profesional, formación de políticas de salud, contribuir a la implementación de continuidad de cuidados, como la fundamentación en un futuro de intervenciones educativas y guías de práctica clínica para el caso particular, aplicadas en los diferentes niveles de atención, con fundamento en la evidencia científica, así como también, contribuir a estudios futuros que profundicen el fenómeno de estudio. Por todo lo anterior señalado, se planteó el siguiente objetivo de investigación: Determinar las interrelaciones existentes entre la Calidad de Sueño, la Adherencia al Tratamiento Terapéutico y los valores de HbA1c en Mujeres con DT2, perteneciente a una comunidad de la ciudad de Puebla.

## Materiales y Métodos

El diseño del estudio fue de tipo descriptivo, correlacional y de corte transversal^16^. La población estuvo integrada por mujeres con DT2 que acudieron a un Centro de Salud del Estado del Puebla, México, perteneciente a una localidad semi urbana. El muestreo fue por conveniencia, debido a que fueron reclutadas las mujeres con DT2 que acudieron a su control glucémico, a la consulta externa del turno matutino, durante los meses de enero a marzo de 2020. La muestra se calculó mediante el paquete estadístico nQuery Advisor® versión 7.0^17^ con un nivel de significancia de .05, un coeficiente de correlación .30 y un poder estadístico del 90%, obteniendo una n= 110 mujeres con DT2^18^.

Se eligieron mujeres de 40 a 69 años que tuvieran diagnóstico médico previo de DT2, que aceptaron participar voluntariamente en el estudio, con firma previa de la hoja de consentimiento informado y un ayuno mínimo de siete horas. Se excluyeron mediante un cuestionario filtro, aquellas mujeres con DT2 que presentaron algunas de las siguientes características: diagnosticadas con algún trastorno del sueño, depresión, ansiedad, obesidad mórbida (IMC > 40 kg/mts2), con antecedente de consumo de psicotrópicos y/o que trabajaran en el turno nocturno. Además de mujeres: embarazadas, diagnosticadas con cáncer y/o anemia. Lo anterior debido a que son factores que modifican la cantidad y calidad de sueño, así como la activación de un proceso neuroendocrino (insulina, cortisol, grelina) y fisiológico que afectan los valores de glucosa en sangre^15^^,^^19^.

La recolección de la información fue mediante entrevistas, se empleó una cédula de datos personales, la cual incluyo datos como: edad, genero, ocupación, años de estudio, estado civil, así como datos respecto a su padecimiento de DT2, medicamentos que toma, signos y síntomas generales de la DT2, tiempo de diagnóstico e Índice de Masa Corporal (IMC), el cual se realizó previa medición de peso y talla, mediante una báscula de impedancia bioeléctrica marca Tanita Ironman Body Composition Monitor, modelo BC-558® y una cinta métrica de mano con valores de referencia en centímetros y milímetros marca HERGOM®. El IMC se obtuvo mediante la fórmula de Quetelet: IMC=Peso (Kg.) / Talla (mts)². Las cifras obtenidas se clasificaron de acuerdo con los criterios de la Norma Oficial Mexicana 008^20^; con obesidad grado I si presentó un IMC ≥ 30 kg/mts2 y en las personas adultas de estatura baja (1.50 mts en mujeres) ≥ 25 kg/mts2, con sobrepeso si presentó un IMC ≥ 25 kg/mts2 y ≤ 29.9kg/mts2; en las personas adultas de estatura baja (1.50 mts en mujeres) ≥ 23 kg/mts2 y ≤ 25 kg/mts2. Se entendió como obesidad grado II, si la mujer presentó un IMC entre 35 y 39.9 kg/m2.

Para conocer la calidad de sueño se utilizó el Índice de Calidad de Sueño de Pittsburgh (PSQI), que consta de 19 preguntas auto aplicadas y cinco preguntas evaluadas por la pareja del paciente o compañero de habitación, mismas que no se puntúan con la escala general, debido a que son referentes sobre el hábito de descanso de la persona. Los 19 ítems autoevaluados se combinan entre sí para formar siete componentes de puntuación: 1.calidad subjetiva, 2.latencia de sueño, 3.duración de sueño, 4.eficiencia de sueño, 5.perturbaciones de sueño, 6.uso de medicación hipnótica y 7.disfunción diurna. Estos se suman para rendir una puntuación global, donde la puntuación mínima es de cero y máxima de 21 puntos, con un punto de corte de cinco puntos, donde a mayor puntaje, no refleja una calidad del sueño. El Índice de Calidad del Sueño de Pittsburgh (PSQI), ha sido utilizado en población mexicana^21^, obteniendo un coeficiente de confiabilidad satisfactorio de 0.78 y coeficientes de correlación significativos de 0.53 a 0.77 entre sus componentes y la suma total. Para la presente investigación el instrumento obtuvo una confiablidad de .71.

Asimismo, se utilizó el Cuestionario de Adherencia Terapéutica MBG, el cual está conformado por 12 preguntas, que conforman tres categorías, 1.cumplimiento del tratamiento; 2.-implicación personal y 3.relación transaccional. Con opción de respuesta en escala Likert compuesta por cinco posibilidades; 4: siempre, 3: casi siempre, 2: a veces, 1: casi nunca y 0: nunca; su interpretación se realiza mediante una sumatoria de todas sus respuestas. El instrumento clasifica como pacientes adheridos totales, aquellos que obtienen una puntuación de 38 a 48, adheridos de forma parcial, de 18 a 37 y no adheridos aquellos de 0 a 17^22^. Este instrumento ha sido aplicado en población mexicana, obteniendo un alpha de Cronbach de .88^23^. Sin embargo, para el presente estudio se obtuvo un valor de .60.

La medición de HbA1c se realizó mediante el dispositivo Eclipse A1c. Los resultados fueron clasificados de acuerdo con la Asociación Americana de la diabetes, con los siguientes puntos de corte: Nivel no diabético: ≤ 5.6%; Nivel prediabético (riesgo aumentado de diabetes o prediabetes): entre 5.7% y 6.4%; Nivel diabético: ≥ 6.5%, (6.5 - 7.9% en control, 8%-10.9% niveles aumentados, 11%-14% niveles severamente aumentados)^24^. La prueba se ejecutó por parte de la investigadora principal y una licenciada en enfermería, con previa capacitación y certificación profesional; las cuales, mediante una punción capilar con previa asepsia y antisepsia del dedo índice, obtuvieron una muestra de 1 mcL de sangre.

El presente estudio se apegó a los principios básicos de bioética y al código de ética de enfermería, así como por lo establecido en el Reglamento de la Ley General de Salud en referencia a los aspectos éticos de la investigación en seres humanos^25^. La investigación fue registrada y avalada por el comité académico de investigación y posgrado, con el número SIEP/ME/074/2019.

Los datos fueron procesados en el Statistical Package for the Social Sciences, versión 24.0 para Windows. Se utilizó estadística descriptiva (frecuencias, porcentajes, medidas de tendencia central, desviación estándar, valor máximo y mínimo). Para dar respuesta al objetivo de investigación, se utilizó estadística inferencial de correlación, ejecutando previamente la prueba de ajuste de bondad de Kolmogorov-Smirnov para determinar la normalidad de las variables, con la finalidad de decidir el uso de la prueba de correlación de Spearman ^26^.

## Resultados

Se encontró una media de edad de 57.67 años (DE = 8.06). El mayor grado de estudios fue de 12 años, con primaria incompleta (44.60%); además de un promedio 8.92 (DE=6.61) años, de haber sido diagnosticadas con DT2 (Tabla 1).

**Tabla 1:** Datos Sociodemográficos de las Mujeres con DT2

Características	Categoría	f	%
Ocupación	Empleada	12	10.91
	Desempleada	4	3.64
	Ama de casa	94	85.45
Estado civil	Soltera	16	14.55
	Casada	48	43.64
	Unión libre	25	22.72
	Viuda	21	19.09
Síntomas	Ninguna	58	52.73
generales	Poliuria	11	10.00
	Polidipsia	8	7.27
	Polifagia	21	19.09
	Todos	2	1.82
	Poliuria y polifagia	6	5.45
	Polidipsia y polifagia	2	1.82
	Poliuria y polifagia	2	1.82
Tratamiento	Hipoglucemiantes	73	66.36
	Insulina	10	9.09
	Ambos	27	24.55
IMC	Normal	17	15.45
	Sobrepeso	50	45.55
	Obesidad grado I	43	39.09

*Fuente*: cédula de datos personales. n: 110

El análisis descriptivo de las demás variables de estudio, se muestran en las Tablas 2 y 3.

**Tabla 2:** Categorización de las Variables de Estudio

Característica	Categoría	f	%
Calidad de	Sin Calidad	66	60.00
sueño	Con Calidad	44	40.00
Adherencia al	Adherencia total	24	21.8 0
Tratamiento	Adherencia parcial	86	78.2 0
Terapéutico			
Valores	Control	52	47. 27
HbA1c	Valores aumentados	44	40.0 0
	Valores severamente aumentados	14	12.7 3

Fuente: cédula de datos personales, PSQY; MGB. n:110

**Tabla 3:** Prueba de Kolmogorov-Smirnov (KS) para las Variables de Estudio

Variable	𝗑	Mdn	DE	Vm	VM	K-S	p
Índice de Calidad de sueño	7.24	7.00	3.60	1	16	.133	.001
Calidad de sueño subjetiva	1.30	1.00	.72	0	3	.352	.001
Latencia del sueño	1.59	1.50	1.19	0	3	.218	.001
Duración del dormir	.78	1.00	.79	0	3	.274	.001
Eficacia del sueño habitual	.90	1.00	.91	0	3	.255	.001
Alteración es del sueño	1.26	1.00	.60	0	3	.342	.001
Uso de medicamentos	.09	.00	.34	0	2	.5 31	.001
Disfunción diurna	1.31	1.00	.94	0	3	.231	.001
Cuestionario MGB	32.47	34.00	5.72	19	42	.114	.001
Cumplimiento al tratamiento	12.27	13.00	2.18	6	16	.150	001
Implicación personal	10.81	11.00	3.03	3	17	.094	.019
Relación transpersonal	9.39	10.00	2.67	0	12	.164	.001
Valores de Hemoglobina glucosilada	8.53	8.00	1.79	6.50	14.00	.176	.001

*Fuente:* cédula de datos personales, PSQY; MGB. n:110

A fin de conocer las estrategias que realizan las mujeres con DT2 para adherirse a su tratamiento terapéutico, se analizaron cada una de las preguntas del cuestionario, encontrando puntuaciones altas para el no cumplimiento con las indicaciones relacionadas con su dieta (f= 76), (69.10%), el nunca realizar los ejercicios físicos indicados (f= 61), (55.50%) y el no utilizar recordatorios que faciliten la realización de su tratamiento (f= 73), (66.40%).

Para dar respuesta al objetivo de investigación, se realizó la prueba de correlación de spearman (rs), debido a que no se demostró normalidad en los datos (p<.05), hallando relaciones estadísticamente significativas entre el índice de calidad de sueño con los valores de HbA1c (rs=-.355; p=.001), los años de diagnóstico con la edad (rs=.360; p=.001) y los años de estudio (rs=-.196; p=.04), no así para la adherencia terapéutica, que no tuvo relación significativa con ninguna de las variables de investigación (p>.05), (Tabla 4).

**Tabla 4:** Matriz de Correlación

Variable	1	2	3	4	5	6	7	8	9	10	11	12	13	14	15	16	17
1 Edad	1																
2 Años de Estudio	-.516**	1															
3 Años de Diagnostico DT2	.360**	-.196*	1														
4 IMC	-.009	.039	-.033	1													
5 Calidad de sueño subjetiva	-.012	-.142	.055	-.010	1												
6 Latencia del sueño	.119	-.192*	-.021	.015	.453**	1											
7 Duración del dormir	.135	-.148	.054	.115	.281**	.406**	1										
8 Eficacia de sueño habitual	.117	-.136	.036	.144	.298**	.413**	.939**	1									
9 Alteraciones del sueño	.064	-.236*	.014	.168	.400**	.422**	.186	.208^*^	1								
10 Uso de medicamentos para dormir	.163	-.054	.070	-.023	.079	.152	.105	.087	.060	1							
11 Disfunción diurna	-.153	.117	-.20*	.002	.276**	.208*	.262**	.295**	.184	.050	1						
12 Índice de calidad de sueño	-.086	.186	.022	-.097	-.632**	-.772**	-.737**	-.755**	-.522**	-.207*	.557**	1					
13 Hemoglobina	.031	-0.073	.091	-.023	-.282**	-.281**	.204*	-.269**	-.099	-.000	-.225*	-.325**	1				
Glucosilada																	
14 Índice de adherencia al tratamiento terapéutico	-.048	.118	.026	-.046	-.046	-.040	-.154	-.152	-.110	-.085	-.145	.148	.102	1			
15 Cumplimiento al tratamiento	.089	.006	.115	-.085	-.109	.096	-.119	-.119	-.094	-.060	-.087	.062	.007	.663**	1		
16 Implicación personal	-.141	.174	-.009	-.085	.045	-.070	-.141	-.121	-.175	-.083	-.144	.145	.154	.798**	.341**	1	
17 Relación transpersonal	-.057	.078	-.004	.116	-.073	-.105	-.121	-.132	.024	-.090	-.022	.120	-.017	.663**	.233*	.281**	1

*Fuente:* cédula de datos personales (n:110), PSQY; MGB; Valor RHO - Correlación de Spearman *p < .05, ** p< .001 e

## Discusión

El presente estudio tuvo como propósito conocer las interrelaciones existentes entre la calidad de sueño, la adherencia al tratamiento terapéutico y los valores de HbA1c en Mujeres con DT2, perteneciente a una comunidad semi urbana de la ciudad de Puebla, México.

Para los datos relacionados con las características de las mujeres con DT2, se coincide con lo mencionado por Castillo^13^, al referir que la mayoría de los participantes del estudio, no identifican los síntomas de poliuria, polidipsia y polifagia, resultado que podría deberse a que no reconocen los signos y síntomas cuando tienen elevada la glucosa, derivado de la poca escolaridad que presentan, así como de los años de padecer la enfermedad, el cual pudieron haberse habituado inconscientemente.

Con respecto a los resultados obtenidos en el índice de calidad de sueño, se concuerda con lo referido por Kodakandla^10^; al encontrar que más de la mitad de las mujeres con DT2, no tienen calidad de sueño. Sin embargo, estos hallazgos se contradicen con lo obtenido por Nefs^8^ y Sakamoto^11^, al referir una mejor calidad de sueño en sus poblaciones de estudio, esto podría deberse a las características socioculturales que presentan sus muestras, realizadas en Países bajos y Japón.

En cuanto a la adherencia al tratamiento terapéutico, los resultados arrojaron que la mayor parte de las mujeres con DT2 tiene adherencia parcial, así como el no “utilizar recordatorios que faciliten la realización del tratamiento”, resultados que coinciden con lo encontrado en el estudio de Castillo^13^; lo que podría indicar una falta de búsqueda de conductas de salud, como consecuencia de los pocos o nulos síntomas que presentan las mujeres con DT2, lo que pudiera hacerles pensar, de que su padecimiento no requiere de mucha atención y cuidados específicos.

Con relación a los valores de HbA1c, se coinciden con lo encontrado por Gozashti^12^, al encontrar cifras por arriba de los valores normales y de control para las personas con DT2. Hallazgo que es consistente con los resultados de adherencia al tratamiento terapéutico; el cual puede derivar en un futuro (si no se corrige a tiempo), a lesiones microvasculares y macrovasculares en la población de estudio.

Se acertó en que la calidad de sueño se relaciona con los niveles de HbA1c, hallazgos que concuerdan con los resultados de Lee^5^, Sakamoto^11^, Gozashti^12^, Siwasaranond^9^, y Kodakandla^10^; lo que confirma de manera indirecta, las reacciones bioquímicas que ocurren durante la privación del sueño, donde paulatinamente hay un incremento en la intolerancia a la glucosa y por consecuencia un aumento del tono simpático, lo que provoca una disminución en la actividad de las células beta del páncreas^14^.

No obstante, es válido mencionar que no se reportó relación significativa de la adherencia al tratamiento terapéutico, con los niveles de HbA1c, resultado que pudo deberse, a la sensibilidad que presento el instrumento en la población de mujeres con DT2, caracterizada por tener una baja escolaridad, así como de otros aspectos socioculturales, familiares y económicos no abordados en el presente estudio. Además de que pudo deberse también a la mala calidad de sueño presentada en la muestra, en donde al no cumplirse con todas las etapas, no se generaron los neurotransmisores responsables de la saciedad: el apetito, el hambre y el gasto energético, generando obesidad, entre otras complicaciones.

Finalmente, los resultados deben considerarse con cautela, debido que solo se limitó a un grupo específico de mujeres con DT2, en un momento en el tiempo. Además de que el instrumento de adherencia al tratamiento terapéutico obtuvo un coeficiente de confiabilidad reservado. Sin embargo, los resultados descubiertos contribuyen al fortalecimiento científico de enfermería, orientando en la mejora de cuidados, que servirá para el diseño de intervenciones que favorezcan a la salud de las mujeres con DT2.

## Conclusiones

El estudio encontró un mayor porcentaje de mujeres que no refieren presentar síntomas de poliuria, polidipsia y polifagia. Más de la mitad presentan obesidad grado I y sobrepeso. El tratamiento que predomino fue el uso de hipoglucemiantes orales.

Finamente, se encontró una relación negativa y significativa entre la calidad de sueño y los niveles de HbA1c; no así para con la variable de adherencia al tratamiento terapéutico, la cual no se encontró ninguna relación estadísticamente significativa con las variables de estudio.
